# Supplementary material for: Aggressive Intravenous Hydration and a Defined Plant-Based Diet Safely and Effectively Treated Type 5 Cardiorenal Syndrome with Stage E Heart Failure-Related Cardiogenic Shock: A Case Report
Source: Reports (MDPI). 2024 Nov 8;7(4):94. doi: 10.3390/reports7040094 (PMC12199980; doi:10.3390/reports7040094)
Supplement: Supplementary file 1 [file reports-07-00094-s001.zip › reports-3268930-supplementary.pdf]

## Supplementary materials

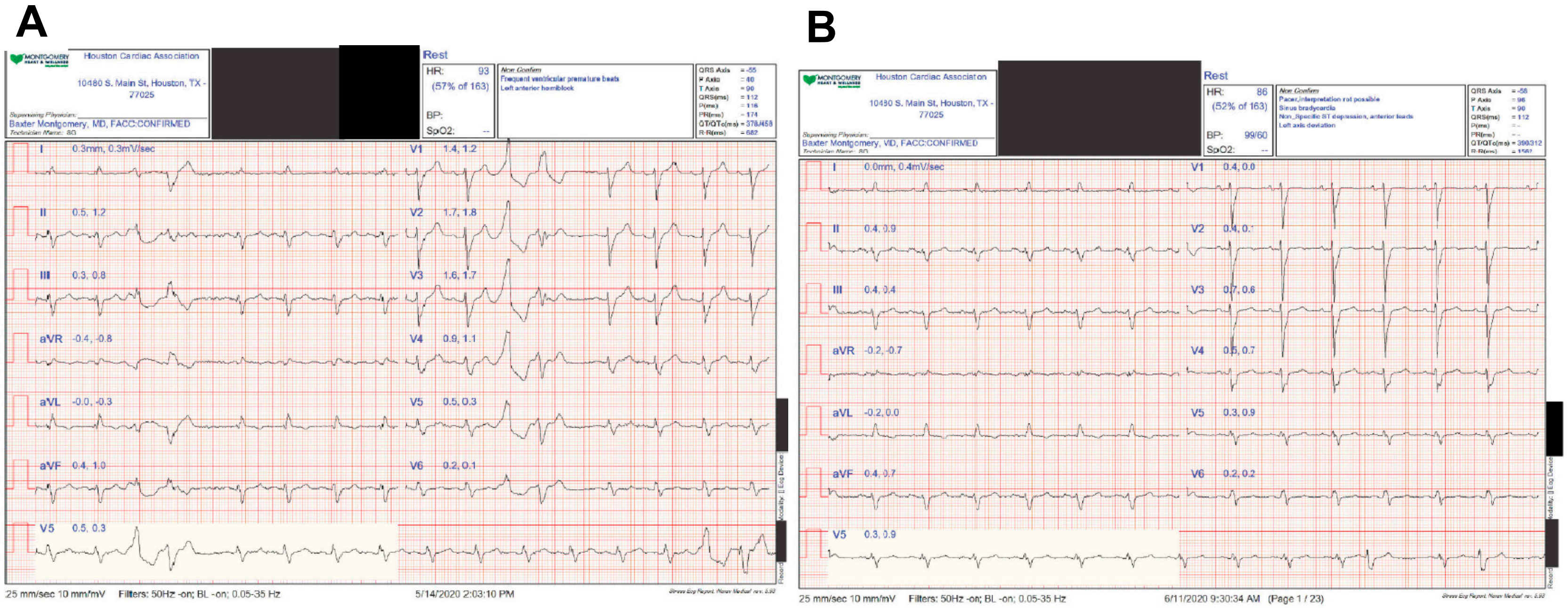


Figure S1. EKG readouts at baseline (A) and following the intervention (B). Identifying information has been redacted.

Table S1. Medication changes

| | **05.14.2020** | **06.12.2020** |
|---|---|---|
| 1 | allopurinol 300 mg once a day | Bystolic 5 mg once a day<br>Vitamin D2 50,000 IU once a week |
| 2 | carvedilol 6.25 mg twice a day | |
| 3 | Tradjenta 5 mg once a day | |
| 4 | Eliquis 5 mg twice a day | |
| 5 | Entresto 97-103 mg twice a day | |
| 6 | torsemide 60 mg twice a day | |
| 7 | atorvastatin 40 mg once a day before bed | |
| 8 | Humalog 15 units as needed with meals | |
| 9 | Lantus 40-80 units once a day | |
| 10 | Vitamin D2 50,000 IU once a week | |
| 11 | Klor-Con M20 20 mEq twice a day | |

Table S2. Additional labs and clinical parameters

| | 5.14.2020 | 5.22.2020 | 5.23.2020 | 5.24.2020 | 5.25.2020 | 5.26.2020 | 5.27.2020 | 5.28.2020 | 5.29.2020 | 6.11.2020 |
|---|---|---|---|---|---|---|---|---|---|---|
| Carbon dioxide (mmol/L) | 18 | 11 | 11 | 18 | 20 | 24 | 26 | 27 | 25 | 26 |
| LVEF (%) | 20% | | | | | | | | | 25% |
| NT pro-BNP (pg/mL) | 1274 | 2772 | | | | | | | 25767 | 5527 |
| Modified Bruce Treadmill Exercise Time (s) | 258 | | | | | | | | | 783 |
| BMI | 32 | | | | | | | | | 30.7 |
| Body weight (kg) | 104.5 | | | | | | | | | 100 |
| Glucose (mg/dL | 158 | 135 | | | | | | | 120 | 140 |
| IV fluids (L) | | 2 | 3 | 4 | 5 | 6 | 7 | 8 | | |
